# Apoptosis in subicular neurons: A comparison between suicide and Addison's disease

**DOI:** 10.4103/0019-5545.58293

**Published:** 2009

**Authors:** K. Printha, S. R. Hulathduwa, K. Samarasinghe, Y. H. Suh, K. R. D. De Silva

**Affiliations:** Genetic Diagnostic and Research Laboratory, Department of Anatomy, Faculty of Medical Sciences, University of Sri Jayewardenepura, Nugegoda, Sri Lanka; 1 Department of Forensic Medicine, Faculty of Medical Sciences, University of Sri Jayewardenepura, Nugegoda, Sri Lanka; 2 Department of Pathology, Faculty of Medical Sciences, University of Sri Jayewardenepura, Nugegoda, Sri Lanka; 3 Department of Pharmacology, College of Medicine, Seoul National University, Seoul, South Korea

**Keywords:** Addison disease, apoptosis, corticosteroids, subiculum, suicide

## Abstract

**Background:**

Stress and depression shows possible links to neuronal death in hippocampus. Subiculum plays a prominent role in limbic stress integration and direct effect of corticosteroids on subicular neurons needs to be defined to assess its subsequent impact on hippocampal plasticity.

**Aim:**

This study was intended to assess apoptosis in subicular neurons of a young depressed suicide victim, where presumably stress induced excess of corticosteroids and a case of young Addison's disease with low level of corticosteroids.

**Materials and Method:**

Both bilateral adrenal glands (Addison's) and subiculum (both cases) were initially stained with hematoxylin and eosin; subicular neurons of both cases were examined for the degree of apoptosis using ‘ApopTag Kit’. Apoptotic cell counts were expressed as average number of labeled cells/mm^2^ and the results were analysed statistically using a non-parametric Mann–Whitney U test.

**Result:**

Apoptotic neurons were detected in the subicular region of both suicide and Addison victims, and it is statistically significant in both right and left between the cases (*P* < 0.05). In suicide victim, the neuronal apoptosis is considerably significant between the two hemispheres (*P* < 0.05), in contrast to Addison disease where the number of neuronal cell death between right and left was statistically insignificant (*P* > 0.05).

**Conclusion:**

The present study confirms the vulnerability of the subicular neurons to apoptosis, possibly due to corticosteroids in both ends of spectrum.

## INTRODUCTION

Sri Lanka has the highest suicide rate in South East Asia, ranking in the first and the eighth positions worldwide with a suicide rate of 47.0 and 23.5 (100 000 population) in 1995 and 2005, respectively.[1] The highest suicidal rate age group since 1950 has shown a shift from older age (above 60 years in 1950s) to 15–29 age group by 1995.[2] Depression plays a major role in suicide and is thought to be involved in approximately 65–90% of all suicide with psychiatric pathologies.[3] Among the patients with corticosteroid-induced psychosis, as many as 33% experience suicidal ideation.[4]

Though the effect of corticosteroids on limbic system have been discussed based on animal models and cell cultures by Sousa and Almeida,[5] which includes mainly the hippocampal sub-regions of dentate gyrus and cornu ammonis, the influence of corticosteroids on subicular neurons and the mechanism underlying the neuronal damage have yet to emerge through autopsy studies as well as in human subjects. Subiculum plays a prominent role in hypothalamo-pituitary-adrenocortical axis and the lesion,[6] and stimulation studies[7] indicate that the hippocampus, acting through the output neurons of ventral subiculum, acts to attenuate the stress-induced glucocorticoid release. Herman and Muller[8] study suggests, based on recent information that the subiculum may also be stress excitatory, and that there may be substantial strain or individual differences in the net contribution of the subiculum, to stress integration. The present study intends to look into the apoptosis on subicular neurons of a depressed young suicide victim, where presumably stress induced excess of corticosteroids and a young case of Addison's disease with low level of corticosteroids.

## MATERIALS AND METHODS

### Depressed suicide victim

A 25-year-old depressed male, who attempted suicide by self-immolation, was admitted to the burns ward with superficial burn injuries involving 81% of the total body surface area. He was uncooperative to treatment, markedly restless during his terminal stage and succumbed to the burn injuries 58 h after admission and the subiculum was collected at 27 h of postmortem interval with the approval of close relatives.

### Addison disease victim

A 32-year-old male clinically presented with characteristic skin hyperpigmentation, weight loss, severe hypotension and the biochemical analysis revealed hyponatremia – 106 mmol/L (reference range: 135.0–148.0 mmol/L) and hyperkalemia - 6.5 mmol/L (reference range: 3.6–5.0 mmol/L). Clinical features were compatible with Addison's disease, and the postmortem findings confirmed the atrophic adrenal cortex, which had a total weight of 2 g (normal – 5 g). Bilateral adrenal glands and the subiculum of the hippocampus were collected 15 h postmortem interval (PMI) with the approval of close relatives.

Formalin-fixed paraffin-embedded tissue blocks of the bilateral adrenal glands (Addison's case) and subiculum (both cases) were initially stained with hematoxylin and eosin (H and E); subicular neurons of both the cases were examined for the degree of apoptosis using ‘ApopTag Plus Peroxidase *In situ* Hybridization and Detection Kit’: an indirect TUNEL assay (Chemicon S7101, CA, USA). Reproducibility, the serial tissue sections were tested repeatedly along with negative and positive control sections. The apoptotic bodies in subicular neurons were identified under light microscope (OLYMPUS, BX 50, Olympus Co. Ltd., Japan) at ×200 magnification and to count the labeled neurons, randomized manual cell counts were carried out. Counting was done using a ×40 objective lens and an ocular graticule with 100 grid squares (1 mm^2^ × 100). Counts were expressed as average number of labeled cells/mm^2^ and the results were analysed statistically using a nonparametric Mann–Whitney U Test.

The study was approved by Ethical Review Committee, Faculty of Medical Sciences, University of Sri Jayewardenepura, Sri Lanka.

## RESULTS

**Figure 1:**
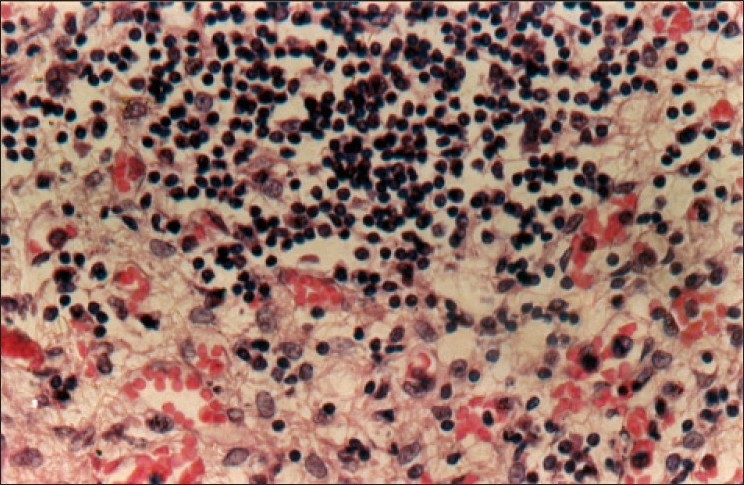
H and E staining of the adrenal gland from the young Addison's disease victim showing heavily infiltrated lymphocytes (×400 magnification)

Histopathological examination of the bilateral adrenal glands from the Addison's case showed an atrophic adrenal cortex and an infiltration by lymphocytes. More than 90% of the adrenal cortex had been destroyed, possibly replaced by dense lymphoplasma cystic infiltration [Figure 1]. Figure 2a and b shows apoptotic bodies in the subicular neurons of both cases and it is statistically significant in both right (*P* = 0.012) and left side (*P* = 0.002) between suicide and Addison's victims [Table 1]. In suicide, the neuronal apoptosis considerably significant between the two hemispheres (*P* = 0.022) in contrast to Addison's subiculum, the number of neuronal cell death between right and left hemispheres was statistically insignificant (*P* = 0.185) [Table 1]. Under H and E staining no necrotic cells were observed in both cases.

**Figure 2a:**
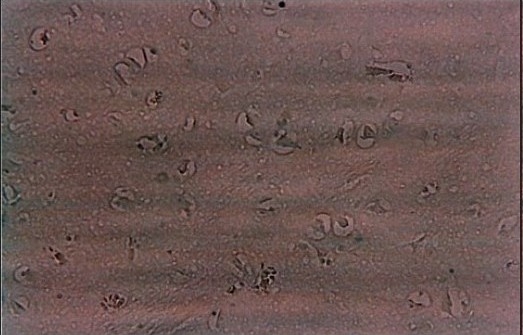
Positive slide of the young suicide victim's subicular neurons to Apop Taq kit (×200 magnification), apoptotic cells are shown by brown color condensed bodies

**Figure 2b:**
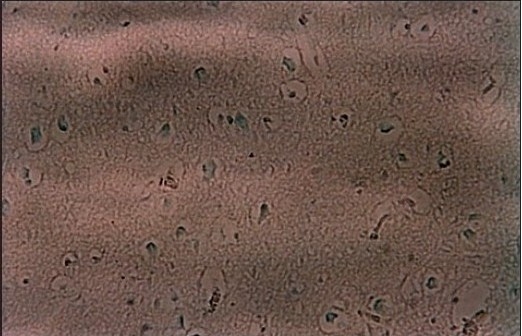
Positive slide of the young Addison's victim's subicular neurons to Apoptaq kit (×200 magnification), apoptotic cells are shown by brown color condensed bodies

**Table 1:** Mann–Whitney U test to apoptotic cell count

Condition	Suicide	Addison's disease	*P* value
Right subiculum (cells/mm^2^)	7.9	3.1	0.012*
Left subiculum (cells/mm^2^)	15.0	5.2	0.002*
*P* value	0.022*	0.185

* Degree of apoptosis was expressed as average number of apoptotic cells per mm^2^ area

## DISCUSSION

It is evident from our study that the subicular neurons of both suicide and Addison's cases contain apoptotic bodies; reflecting the possible underlying mechanism of the neuronal damage is apoptosis in humans. The revised ‘de Kloet's Pendulum Hypothesis’ on hippocampal cell morphology describes, both the absence of corticosteroids (example through adrenalectomy) and activation of glucocorticoid receptors (example via dexamethasone) leads to neuronal cell death.[5] The activation of glucocorticoid receptors leads to hippocampal cell death most probably through apoptosis, while the mineralocorticoid receptors trigger a neuroprotective mechanism, which may be adequate to overcome the death associated with glucocorticoid receptor activation. This predicates the adverse effects of excess corticosteroids on hippocampal cell morphology and it could be reversible with time through the protective action of mineralocorticosteroid. In Addison's case, the level of mineralocorticosteroid is possibly low (level was not recorded) due to atrophic adrenal cortex. As per se, in the studies of Sousa and Almeida,[5] Crochemore *et al*,[9] there may be a lack of neuroprotective effect of mineralocorticosteroid receptor resulting in the left and the right subicular neurons being affected in a similar manner (*P* = 0.185). In contrast, to the suicide, apoptotic neuronal death is significantly higher in the left compared to the right subiculum (*P* = 0.022). It is in accordance with Neveu *et al*,[10] study on mice, which confirms regardless of behavioral lateralization, there is a tendency for right dominance in mineralocorticosteroid receptor binding capacity in contrast to glucocorticosteroid receptor binding capacity, which is similar in each hemisphere, explaining the possibility of low neuronal loss in the right side of the suicide case.

In conclusion, the present study confirms the vulnerability of the subicular neurons to apoptosis possibly due to changes in corticosteroids in both ends of a spectrum.

### Limitations

Our study does have some potential shortcomings. The small sample size (only two brains were collected at autopsies in Colombo) may result in diminished statistical power to conclude the subicular neuronal vulnerability to apoptosis in both ends of a spectrum. Collecting young suicide brain is a difficult target due to judicial and other legal formalities. Similarly getting Addison's disease brain from a young case also a rare occurrence as the prevalence of Addison's disease among the people comparatively low in numbers (39–60 per million of the population).[11] The level of the corticosteroids in suicide case and mineralocorticosteroid in Addison's case were not recorded as the routine government hospitals do not have such facilities. Though the commercially available, more reliable and reproducible techniques are used to identify and quantify the apoptotic bodies in tissue sections, they can also produce high background and false-positive staining, which renders the distinction between apoptosis and necrosis difficult.[12–14] Terminal deoxynucleotidyl transferase enzyme in the ApopTaq kit method labels the free 3′ ends generated by the characteristic endonuclease cleavage of DNA during apoptosis. This is difficult in human postmortem brain tissue, as obstructions in controlling variables related to the quality of tissue preservation; low tissue pH due to antemortem hypoxia, postmortem autolysis, archival length in buffered formalin and other factors may cause DNA fragmentation.[15,16]
